# Novel Virtual Screening Approach for the Discovery of Human Tyrosinase Inhibitors

**DOI:** 10.1371/journal.pone.0112788

**Published:** 2014-11-26

**Authors:** Ni Ai, William J. Welsh, Uma Santhanam, Hong Hu, John Lyga

**Affiliations:** 1 Pharmaceutical Informatics Institute, College of Pharmaceutical Sciences, Zhejiang University, Hangzhou, Zhejiang, P.R. China; 2 Department of Pharmacology, Robert Wood Johnson Medical School, Rutgers University, Piscataway, New Jersey, United States of America; 3 Global R&D, AVON Products, Inc., Suffern, New York, United States of America; Stanford University, United States of America

## Abstract

Tyrosinase is the key enzyme involved in the human pigmentation process, as well as the undesired browning of fruits and vegetables. Compounds inhibiting tyrosinase catalytic activity are an important class of cosmetic and dermatological agents which show high potential as depigmentation agents used for skin lightening. The multi-step protocol employed for the identification of novel tyrosinase inhibitors incorporated the *Shape Signatures* computational algorithm for rapid screening of chemical libraries. This algorithm converts the size and shape of a molecule, as well its surface charge distribution and other bio-relevant properties, into compact histograms (signatures) that lend themselves to rapid comparison between molecules. *Shape Signatures* excels at scaffold hopping across different chemical families, which enables identification of new actives whose molecular structure is distinct from other known actives. Using this approach, we identified a novel class of depigmentation agents that demonstrated promise for skin lightening product development.

## Introduction

Melanin, which is widely distributed in the plant and animal kingdom is responsible for the undesirable browning of fruits and vegetables, as well as the development of skin, hair and eyes coloring in animals [1],[2]. Melanin is produced by melanocytes through the conversion of the amino acid L-tyrosine to 3,4-dihydroxyphenylalanine (L-DOPA) which is then oxidized to yield dopaquinone [3]–[5], the precursor for melanin formation. Tyrosinase is a multifunctional copper-containing enzyme that catalyzes the rate-limiting step for melanin biosynthesis [6],[7]. This tyrosinase-catalyzed process is also involved in abnormal accumulation of melanin pigments that leads to dermatological hyperpigmentation disorders [6]. Therefore, tyrosinase inhibitors such as kojic acid and arbutin have been established as important constituents of cosmetic products for skin whitening and the depigmenting agents for hyperpigmentation [8]. Likewise, there is increasing recognition of the importance of tyrosinase inhibitors in the food industry as well as in medicinal and cosmetic products. Several recent review articles provide a comprehensive summary about the currently available tyrosinase inhibitors from synthetic, semi-synthetic and natural origin [9],[10].

*Shape Signatures*, a novel virtual screening approach, has demonstrated its utility for identification of lead compounds in previous molecular discovery studies [11]. The *Shape Signatures* algorithm fully explores the three-dimensional volume of the molecule, producing a compact histogram representation that encodes its molecular size, shape and surface charge distribution. Large commercial organic compound libraries, up to millions of compounds, from multiple sources can be processed through the algorithm rapidly and stored as S*hape Signatures* databases for future use over and over again. For an identified compound of interest with known activity (the query), *Shape Signatures* compares the query's histogram with the corresponding histograms of pre-generated *Shape Signatures* databases to identify potential hits with similar shape to the query compound. The underlying premise is that these hits would perform similarly as the query compound in the biological system. Each hit is ranked in order of similarity to the query, and assigned a similarity score using one or more simple metrics [11]–[15].

In this study, we adopted *Shape Signatures* within a multi-step scheme to screen chemical libraries of compounds as potential tyrosinase inhibitors for cosmetic purposes.

## Materials and Methods

### Virtual Screening Procedures

Two prototypical tyrosinase inhibitors, viz., kojic acid and glabridin, were selected as queries for the present study. The three-dimensional conformations of these two compounds were generated using the program CORINA (Molecular Networks GmbH) with default settings, saved as mol2 files, and uploaded to our in-house *Shape Signatures* server.

Each query molecule was converted to its corresponding shape signature as described previously [11]. The customized ray-tracing algorithm explored the molecular volume (bound by the solvent accessible surface) by determining the lengths of 100,000 ray segments using the laws of optical reflection inside the triangulated surface. The ray-segments were then sorted into bins, yielding a histogram representing that specific molecule's one-dimensional (1D) shape signature.

The compounds examined for tyrosinase inhibitory effect were the>200,000 commercially available organic compounds marketed by Aldrich, Asinex, Bionet, LeadQuest, Maybridge, and InterBioScreen. These compounds had already been converted to their corresponding shape signature representations in preparation for previous studies, thus no further preparation of the data base was necessary prior to the present screening for tyrosinase inhibitors [12]–[15]. The histograms of the query and data base molecules were compared rapidly using the chi-square (χ^2^) metric. The deviation between the histograms provided a dissimilarity score Δ for the two molecules being compared. A lower score indicated greater similarity between the two molecules, such that Δ = 0.00 denotes identity.

A subset of 200 compounds was selected from the libraries by combining the 100 top-scoring hits for each of the two queries (kojic acid and glabridin) based on their 1D shape signature scores. These 200 hits represented by MDL MACCS structural keys were grouped into 10 structurally distinct classes according to their pair-wise Tanimoto distances using the Jarvis-Patrick clustering method within the MOE program (Chemical Computing Group Inc., Montreal CA). Details of the procedure are provided elsewhere [15].

In order to evaluate the relative binding affinity of the hit compounds to the human tyrosinase, and in the absence of a high-resolution X-ray crystal structure in the Protein Data Bank for human tyrosinase at the initiation stage of this work, a three-dimensional structural model of this enzyme was built using computational homology modeling methods. The protein sequence of human tyrosinase (accession no. AAK00805) was retrieved from the National Center for Biotechnology Information Reference Sequence (RefSeq) Collection. A structural model of the catalytic domain of tyrosinase was constructed using the Insight II Homology Module (Accelrys, Inc., San Diego, CA) from the published crystal structure of a plant catechol oxidase [16] (RCSB Protein Data Bank [17]; PDB ID = 1BUG) as the modeling template. The overall quality of the model was confirmed by the WHATIF-Check program. The crystal structure of human tyrosinase was published during the process of this work. The root-mean-square-deviation value (RMSD) between homology model and newly available crystal structure of human tyrosinase was calculated for heavy atoms in the enzyme to evaluate the quality of homology model (RMSD<1.5 Å) in terms of deviation between C_α_ atoms of the two structures.

**Figure 1:**
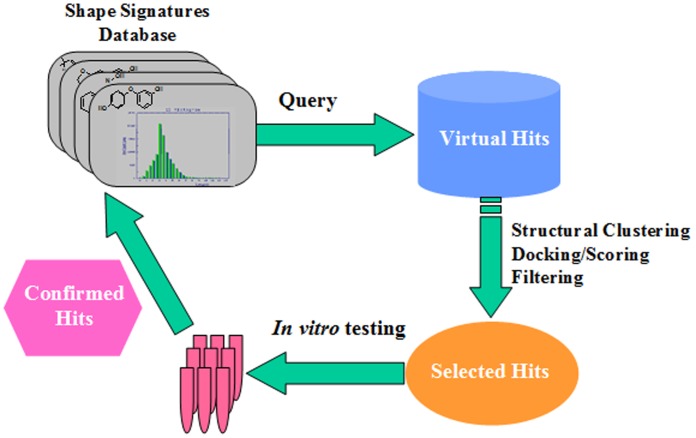
Overview of computational procedure employed in this study to identify potential tyrosinase inhibitors. Experimentally positive virtual hits are fed back to the server and the process is repeated iteratively to achieve the optimum lead compound.

Representative compounds (the center compound in each cluster) from each of the 10 clusters were docked inside the structural model of tyrosinase catalytic pocket using the ligand-receptor docking program GOLD (Genetic Optimization of Ligand Docking) [18]. The intermolecular interactions between the two queries (kojic acid and glabridin) and human tyrosinase were employed as reference points for the docked test compounds. The top-ranked conformation of each docked test compound was selected from 30 independent dockings, and the corresponding GOLD score and three contributing components of the overall GOLD score were employed as filters to select promising compounds for biological evaluation. A schematic of the general screening procedure is depicted in **Figure 1**. Substructure searching on *Lead1* was carried out through NCBI PubChem website.

### Testing Compounds

The testing compounds were acquired from the following venders: kojic acid (positive control) and *cmpd 1* (Sigma-Aldrich, St.Louis, MO); *cmpd 2* (Asinex Corp., Winston-Salem, NC); *cmpd 3* and *cmpd 4* (Bionet, KeyOrganics Ltd., Camelford, Cornwell, UK); and *cmpd 5* (Maybridge, Ryan Scientific Inc., Isle of Palms, SC). *Lead1*, its analogues A1–A4, and *Lead2* were purchased from Bionet (KeyOrganics Ltd., Camelford, Cornwall, UK). A stock solution of each compound was prepared in methanol; the concentration of methanol in the final aqueous assay solution was less than 2.5%.

### Mushroom Tyrosinase Inhibition Assay

Mushroom tyrosinase and L-Tyrosine were obtained from Sigma-Aldrich, Inc. (St. Louis, Mo.). The enzyme activity was measured in buffer containing 100 mM phosphate buffer pH 6.8, 5% absolute ethanol, 2 µg/ml mushroom tyrosinase, and 0.2 mg/ml L-Tyrosine. The reaction (conversion of L-Tyrosine to DOPAchrome) was conducted at 25°C for 30 min, and absorbance was then measured at the wavelength of 500 nm. The inhibition assays were carried out in the presence of the test compound at 10 µM concentration for initial screening. Kojic Acid was used as a positive control inhibitor in these assays.

### Melanin Biosynthesis Assay

The B16 cells were seeded into 96-well tissue culture-treated plates and treated with test actives diluted in DMEM without phenol red and examined for their ability to modulate pigment formation. Kojic acid was used as the positive control inhibitor. Cells were exposed to diluted test material or controls for 7 days. Following the treatment period, the level of pigment produced or melanin synthesized was quantified by reading the absorbance at 540 nm using a standard microplate reader (Tecan Group Ltd.). After quantifying the amount of melanin, cell viability was determined using the MTT conversion method. The MTT conversion method measures the reduction of the MTT dye from a yellow colored, water-soluble, tetrazolium salt to a bluish-purple colored insoluble formazan precipitate by NAD(P)H-dependent microsomal dehydrogenase enzymes, which only function in viable cells. The intensity of the blue color is indicative of cell viability. After quantifying the amount of melanin pigment produced, the cells were exposed to MTT dye solution (1 mg/ml) for two to three hours. Formazan material was solubilized with reagent alcohol (95% ethanol: 5% isopropanol) and shaken on an orbital shaker for 15–30 minutes. MTT dye uptake and conversion by viable cells were determined by measuring the extracted formazan at 570 nm using a microplate reader. Total pigmentation was calculated, normalized to cell viability values and expressed as percent activity relative to control.

## Results and Discussion

To identify small molecules with favorable inhibitory activity on human tyrosinase, we searched our *Shape Signature* databases converted from multiple vendor compound libraries that contained>200,000 non-redundant and chemically diverse compounds. As commercially available compounds, all of them were easily obtainable for laboratory testing. In this study, a multi-step virtual screening protocol was created for the analysis of a large database of compounds. The top-scoring 200 matches, 100 from each of the two queries kojic acid and glabridin, were retrieved from Shape Signature searching. The 200 matches were partitioned into 10 classes based on chemical similarity index using Jarvis-Patrick clustering analysis. One representative compound from each cluster was selected for further evaluation. This decision economized on the more time-intensive subsequent steps of ligand-receptor docking and biological evaluation.

As part of our docking studies, multiple scoring and filtering steps were applied to maximize the accuracy and value of the output scores. The optimal parameters of the scoring and filtering steps were determined by a calibration set, consisting of the two query molecules kojic acid and glabridin and another four well-known tyrosinase inhibitors with different inhibition potencies. Each of the molecules in the calibration set were manually docked into the catalytic pocket of the human tyrosinase structure model, and the overall GOLD docking score and three contributing components were computed as summarized in **Table 1**. The docking studies indicated that aloesin had the lowest (i.e., poorest) overall GOLD score (25.10) among the calibration set molecules. Therefore, the value of 26.00 was set as the low threshold for the GOLD score in the screening procedure. In addition to overall GOLD score, three additional key component terms of the GOLD score indicated common features for good tyrosinase inhibitors: 1) interaction with the copper ion in the dinuclear center (S_metal_>1.00); 2) attractive lipophilic interactions (S_lipo_>65.00); and 3) minimal steric clash terms (DE_clash_<5). Any compound failing to satisfy even one of these thresholds was discarded. With these defined criteria, the screening process retrieved several hits with high affinity to the human tyrosinase. The overall GOLD score and the three component terms for the five selected hits are shown in **Table 1**. The values of these four terms for these five hits equaled or exceeded the corresponding values for the two query molecules; therefore, they were selected for further biological evaluation as potential human tyrosinase inhibitors.

**Table 1:** Overall GOLD score and 4 component terms for the calibration set, selected hits and two lead compounds.

Compound	GOLD Score	S_metal_	S_lipo_	DE_clash_
**Calibration Set**				
Kojic Acid	29.51	2.99	64.93	4.89
Glabridin	34.34	1.97	171.41	2.77
Aleosin	25.10	1.90	172.96	8.16
4-hydroxy chalcone	32.20	2.00	143.70	1.89
Hydroxy quinone	26.62	1.97	85.39	3.80
Hydroxy stilbene	33.97	1.97	147.94	2.73
**Selected Hits**				
*Cmpd1*	36.27	2.99	166.03	1.91
*Cmpd2*	31.92	1.00	123.20	2.40
*Cmpd3*	41.70	3.22	111.28	1.95
*Cmpd4*	29.18	2.99	95.14	1.98
*Cmpd5*	33.01	3.94	84.83	1.95
**Lead Compounds**				
*Lead1*	42.39	2.99	219.95	4.35
*Lead2*	37.23	2.99	170.15	2.43

To experimentally validate the *in silico* predictions, we obtained test samples for each of the five hit compounds and assayed them for their ability to inhibit mushroom tyrosinase activity in a cell-free in vitro assay. The tyrosinase activity was suppressed by all five hits. Specifically, *cmpd1* and *cmpd2* exhibited exceptionally high tyrosinase inhibition activity with over 90% inhibition at 10 µM concentration (91.3%±3.35% cmpd1, and 93.2%±6.8% *cmpd2*; n = 3). Compound *cmpd4* showed appreciable inhibitory effect (77%±3.63% inhibition at 10 µM; n = 3) in this assay. The other two hits, *cmpd3* and cmpd5, exhibited moderate inhibition activity (36%±6.54% and 43%±3.3%, respectively; n = 3). Kojic acid, commonly used as a reference compound, is reported in the literature to inhibit 38% tyrosinase catalytic activity at 10 µM concentration. The present virtual screening protocol identified several hits, exemplified by *cmpd1*, *cmpd2*, and *cmpd4*, that exhibited appreciably stronger tyrosinase inhibition than the reference compound kojic acid.

The inhibitory effects of the five hit compounds on melanin biosynthesis in B16 melanoma cells were also examined. However, the melanin production in B16 melanoma cells was not affected by the treatment with each of the five hits. This result suggested there are some issues related to cell penetration for these compounds. The potent inhibitory activity of our hits against tyrosinase *in vitro* motivated us to perform further virtual screening to identify hits with good tyrosinase inhibiting effects and the ability to suppress melanin production in B16 cells.

**Figure 2:**
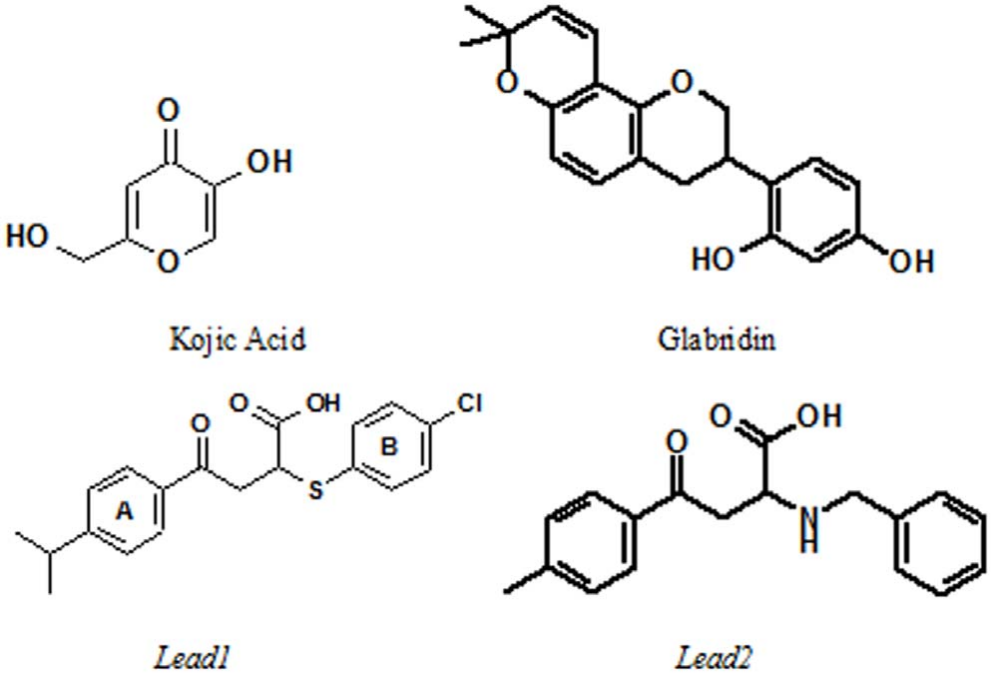
Chemical structures of two lead compounds obtained from our virtual screening procedure and two query molecules, kojic acid and glabridin, used to screen the resident database.

**Figure 3:**
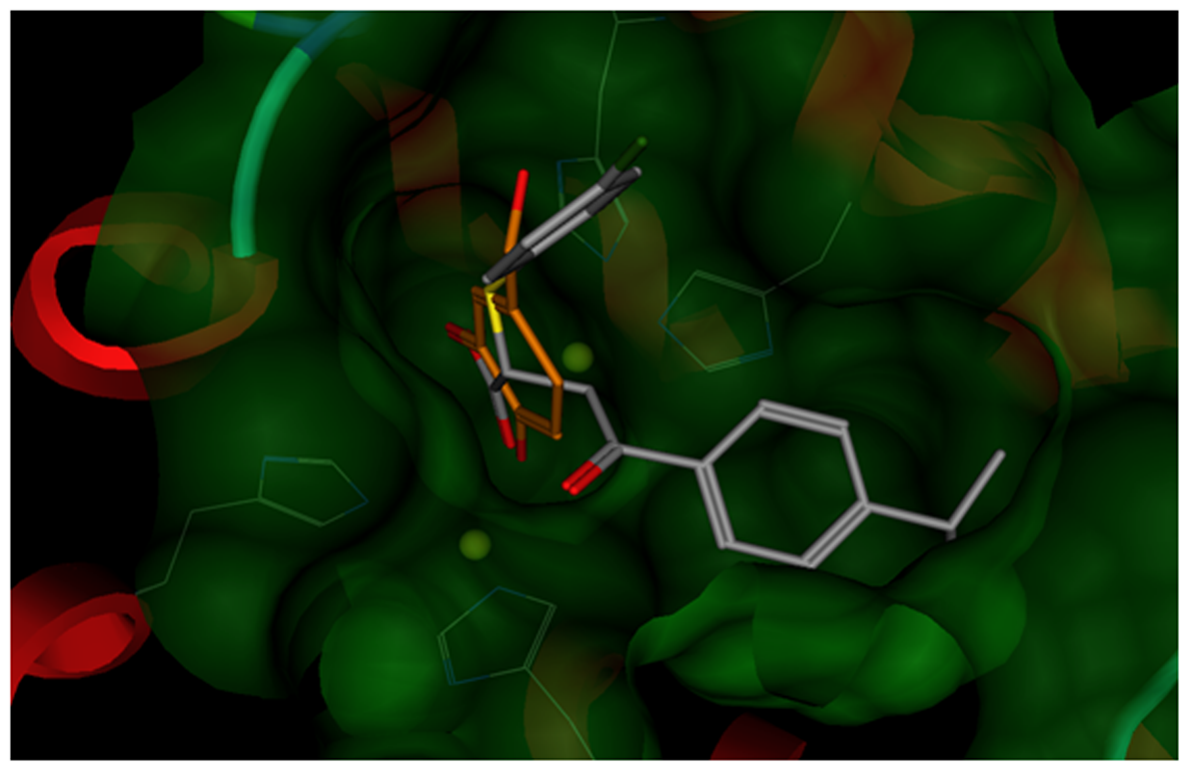
Molecular modeling studies of tyrosinase-ligand complex. The binding orientations of query molecule kojic acid and *Lead1* were displayed. The copper ions were represented as yellow balls. The atoms in the ligands were colored by atom types (Oxygen: red, Nitrogen: blue, Sulfur: bright yellow, Copper: dark green, and Carbon: grey in lead1 and orange in kojic acid). The histidine residues interacting with copper ions are shown also. The Connolly surface of catalytic site of tyrosinase was mapped in transparent green.

Two of the first-round hits, *cmpd1* and *cmpd2*, were then submitted as query molecules for a second round of screening using the same multi-step protocol. This process led to a compound *Lead1* with appreciable *in vitro* activities. The enzyme inhibition assay indicated that *Lead1* inhibited the catalytic activity of mushroom tyrosinase with an IC_50_ value of 8 µM and yielded a 29%±17.64% (n = 3) blockage of melanin biosynthesis in B16 cells at a concentration of 0.002% that was equal to 27.5 µM. The chemical structures of the two original queries (kojic acid and glabridin) together with *Lead1* are depicted in **Figure 2**. Kojic acid and glabridin appear to form the critical interaction with the copper ions in the di-nuclear catalytic center of tyrosinase via their common hydroxyl groups, in the same manner as the substrate L-DOPA. In *Lead1*, this interaction is provided by its carboxylate group serving as a metal chelator. Docking studies confirmed this hypothesis, as evidenced by the close proximity between of the copper ions of tyrosinase and either the carboxylate group of *Lead1* or the hydroxyl group of kojic acid and glabridin (**Figure 3**). In addition to its electrostatic interactions with the copper ions, *Lead1* exhibited extensive van der Waals interactions with the enzyme. Notably, its B-ring occupied a hydrophobic pocket nearby the di-nuclear center which contributes significantly to the lipophilic term of its GOLD score (as shown in **Table 1**). Moreover, *Lead1* is oriented in a similar manner deep inside the catalytic site, occupying the same part of the binding pocket as the native substrate. The functional groups in *Lead1* and L-DOPA differ appreciably. It is likely that *Lead1* would have eluded selection for biological testing using traditional metrics for structural similarity such as MACCS fingerprints. Consistent with our previous studies with *Shape Signatures*, its inclusion in our virtual screening protocol is seen to facilitate scaffold hopping thereby promoting the discovery of hits that possess similar biological profiles, but different chemical structures, from the query molecule.

**Figure 4:**
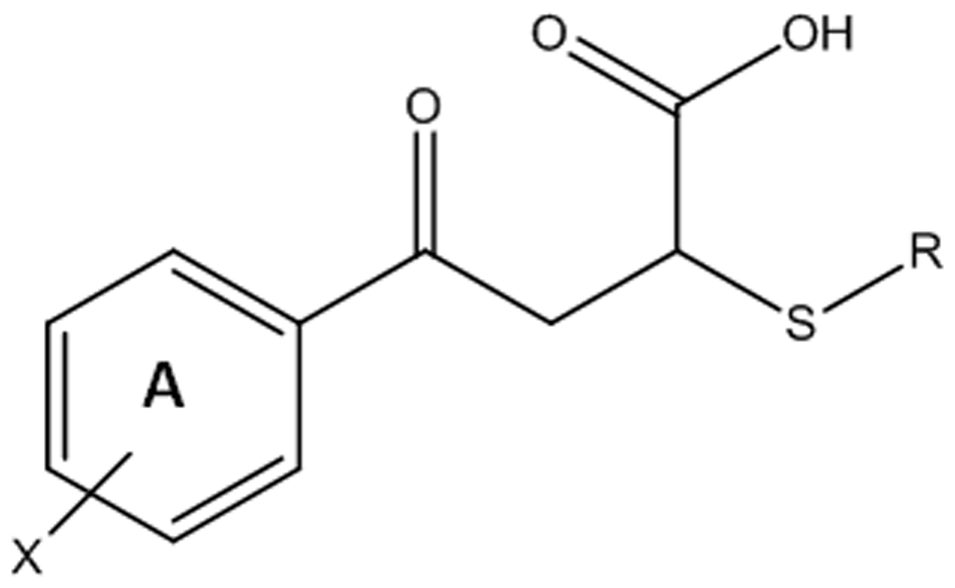
Generic structure of Lead1 analogues.

A limited structure-activity relationship study was conducted around the scaffold of *Lead1* (shown in **Figure 4**). **Table 2** shows the inhibitory activity of the four *Lead1* analogues on melanin biosynthesis in B16 cells. All four analogues exhibited potent inhibitory effects in this assay. The aromaticity of the B ring of *Lead1* appears essential for activity, which was demonstrated by the loss of inhibitory activity by replacing the B ring with a saturated cyclohexyl ring. The substitution on the 4-position of the A ring had a negligible effect on the inhibitory activity of this series of compounds. To demonstrate that these cellular effects are dose dependent, these compounds were prepared and evaluated again at lower concentrations. The results indicated that lower concentration significantly decreased their ability to inhibit melanin production in B16 cells (**Table S1**). During further development of *Lead1*, a potential formulation problem caused this compound to be eliminated as a candidate for cosmetic purposes. A substructure search was performed to address this problem, leading to *Lead2* (shown in **Figure 2**). This compound exhibited 79%±5.34% (n = 8) inhibition on melanin biosynthesis of B16 cells at a concentration of 0.001% (33.6 µM).

**Table 2:** Activity of *Lead1* analogues on melanin biosynthesis in B16 cells.

Compound	X	R	B16 inhibition at 0.001% n = 6	Molarity of 0.001%
**A1**	4-ethoxy	4-chlorobenzyl	85%±0.94%	26.39 µM
**A2**	4-t-butyl	cyclohexyl	47%±5.33%	28.73 µM
**A3**	4-chloro	4-chlorobenzyl	78%±5.22%	27.1 µM
**A4**	4-chloro	cyclohexyl	56%±10.55%	30.67 µM

Virtual screening approaches are common practice for lead identification in drug discovery campaigns [19]–[21]. However, molecular docking studies of the extensive libraries of compounds are often tedious and time consuming. In contrast, the multi-step screening protocol described herein employed a fast ligand-based molecular shape comparison algorithm, *Shape Signatures*, in the first step to select an initial hit list of compounds based on molecular similarity to the query molecule. This measure eliminates the majority of the compounds in the databases. Subsequent clustering of the remaining hits compounds and selection of representative hits from each cluster further reduced the hit list to a tractable number of compounds for the docking studies. Altogether, the subject virtual screening protocol dramatically reduces the computation time and affords rapid and efficient screening of large databases. *Shape Signatures* uniquely matches molecules based on similarity in size, shape, and electrostatic surface features rather than on chemical structure, hence it excels in scaffold hopping [22],[23]. This strategy represents a powerful tool for lead discovery and optimization that yields molecules with new chemistries.

The first generation and second generation hits identified in this study are novel structures that can provide new insights into the tyrosinase catalytic process. *Lead1* and *Lead2* are highly promising candidates for further analysis and development. They possess good physiochemical properties and satisfy Lipinski's “rule of five”[24]. In addition, our experiments confirmed that these two lead compounds exhibited a substantial inhibitory effect on melanin biosynthesis in B16 cells. This melanin biosynthesis inhibition was shown not to affect cellular viability (data not shown), which further underscores the potential commercial utility of these compounds.

## Conclusions

This study introduces a fast and efficient virtual screening protocol that herein led to the discovery of previously unrecognized tyrosinase inhibitors. It was accomplished by virtual screening for inhibitors of tyrosinase using the *Shape Signatures* tool together with established clustering, homology modeling, and ligand-receptor docking methods. This multi-step procedure resulted in two lead compounds that were experimentally confirmed as potent tyrosinase inhibitors [25]. The experimental data obtained provides a strong indication of the usefulness of our novel screening and docking approach.

## Supporting Information

Table S1 Dose dependent inhibition of melanin biosynthesis in B16 cells by *Lead1* analogues.
(DOCX)
