# Supplementary material for: Novel Virtual Screening Approach for the Discovery of Human Tyrosinase Inhibitors
Source: PLoS One. 2014 Nov 26;9(11):e112788. doi: 10.1371/journal.pone.0112788 (PMC4245091; doi:10.1371/journal.pone.0112788)
Supplement: Table S1 — Dose dependent inhibition of melanin biosynthesis in B16 cells by Lead1 analogues. (DOCX) [file pone.0112788.s001.docx]

**Table S1.** Dose dependent inhibition of melanin biosynthesis in B16 cells by *Lead1* analogues.

|  | **Concentration** | **% change** | **SD** | **n** |
| --- | --- | --- | --- | --- |
| A1 | 0.001% | 85.12% | 0.94% | 6 |
|  | 0.0002% | 22.24% | 20.92% | 6 |
| A2 | 0.001% | 47.33% | 5.33% | 6 |
|  | 0.0002% | 0.55% | 3.97% | 6 |
| A3 | 0.001% | 78.02% | 5.22% | 6 |
|  | 0.0002% | 7.16% | 8.85% | 6 |
| A4 | 0.001% | 56.14% | 10.55% | 6 |
|  | 0.0002% | 12.32% | 29.01% | 6 |
| Kojic Acid | 0.010% | 76.19% | 3.84% | 6 |
|  | 0.0010% | 31.46% | 25.15% | 6 |
| Licorice Extract | 0.001% | 63.27% | 1.76% | 6 |
